# Are Portuguese Cowpea Genotypes Adapted to Drought? Phenological Development and Grain Quality Evaluation

**DOI:** 10.3390/biology12040507

**Published:** 2023-03-27

**Authors:** Rita Moreira, Cátia Nunes, Isabel P. Pais, José Nobre Semedo, José Moreira, Ana Sofia Bagulho, Graça Pereira, Maria Manuela Veloso, Paula Scotti-Campos

**Affiliations:** 1 Unidade de Biotecnologia e Recursos Genéticos, Instituto Nacional de Investigação Agrária e Veterinária, I. P., Av. República, 2784-505 Oeiras, Portugal; 2 Unidade de Geobiociências, Geoengenharias e Geotecnologias (GeoBioTec), Faculdade de Ciências e Tecnologia (FCT), Universidade NOVA de Lisboa (UNL), Monte de Caparica, 2829-516 Almada, Portugal; 3 Instituto Nacional de Investigação Agrária e Veterinária, I. P., Estrada Gil Vaz, Ap. 6, 7350-901 Elvas, Portugal

**Keywords:** cowpea, drought, food security, grain filling, landraces, quality and nutritional traits

## Abstract

**Simple Summary:**

The Mediterranean region is highly vulnerable to climate change, with more prolonged and intense droughts threatening food security and biodiversity. Genetic diversity can provide plant material for breeders to develop crops capable of surviving in adverse conditions while producing nutritious and healthy foods. Cowpea [*Vigna unguiculata* (L.) Walp.] is a nutritionally rich legume generally considered drought tolerant. Portugal has a vast diversity of cowpea landraces that may contribute to more sustainable agrosystems; however, they remain largely unexplored. Five cowpea genotypes from different regions of the country were evaluated to understand plants’ responses to terminal drought at a phenological level. The physical and nutritional parameters of the produced grains were also assessed. Results indicate early maturation as a strategy to avoid drought in some of these varieties, combined with significant changes in the aerial part of the plants, such as reductions in the number of leaves, flowers, and fruits. Grain quality parameters also revealed adaptation to low water availability environments, with little variations found except for levels of sugars from the raffinose family. No changes were observed in grain weight, grain color, or protein content. This study reveals some fundamental traits of these genotypes to cope with drought and preserve grain quality and their importance in integrating breeding programs.

**Abstract:**

Along with population growth, global climate change represents a critical threat to agricultural production, compromising the goal of achieving food and nutrition security for all. It is urgent to create sustainable and resilient agri-food systems capable of feeding the world without debilitating the planet. The Food and Agriculture Organization of the United Nations (FAO) refers to pulses as a superfood, as one of the most nutritious crops with high health benefits. Considered to be low-cost, many can be produced in arid lands and have an extended shelf-life. Their cultivation helps reduce greenhouse gases and increases carbon sequestration, also improving soil fertility. Cowpea, *Vigna unguiculata* (L.) Walp. is particularly drought tolerant, with a wide diversity of landraces adapted to different environments. Considering the importance of knowing and valuing the genetic variability of this species in Portugal, this study assessed the impact of drought on four landraces of cowpea (L1 to L4) from different regions of the country and a national commercial variety (CV) as a reference. The development and evaluation of morphological characteristics were monitored in response to terminal drought (imposed during the reproductive phase), and its effects were evaluated on the yield and quality of the produced grain, namely the weight of 100 grains, color, protein content, and soluble sugars. Under drought conditions, the landraces L1 and L2 showed early maturation as a strategy to avoid water deficit. Morphological alteration of the aerial part of the plants was evident in all genotypes, with a rapid reduction in the number of leaves and a reduction in the number of flowers and pods by between 44 and 72%. The parameters of grain quality, the weight of 100 grains, color, protein, and soluble sugars did not vary significantly, except for sugars of the raffinose family that is associated with the adaptive mechanisms of plants to drought. The performance and maintenance of the evaluated characteristics reflect the adaptation acquired in the past by exposure to the Mediterranean climate, highlighting the potential agronomic and genetic value, still little exploited, that could contribute to production stability, preserved nutritional value, and food safety under water stress.

## 1. Introduction

Pulses belong to the Fabaceae (or Leguminosae) family, the third largest on the planet, with about 800 genera and 18,000 to 19,000 species [1]. Pulses are an integral part of human and animal food, being today important crops in the fight against malnutrition and poverty and the quest for a healthier and environmentally friendlier diet. Additionally, pulses contribute to the maintenance and improvement of ecosystems [2], thus fitting in the three pillars of sustainable development [3].

Cowpea (*Vigna unguiculata* (L.) Walp), in particular, is an annual herbaceous legume native to Africa and widely distributed throughout the world. Close to nine million tons of grain are produced per year, resulting from the cultivation of 15 million hectares of land around the world, data from 2020 [4]. In addition to human food, cowpea is used in animal feed, as fodder, also serving as green manure (harvest residues, roots, and stems provide organic matter and nutrients to the soil) and soil cover [5], suppressing weed growth, providing protection against soil erosion and reducing soil temperature. It is globally considered to be tolerant to most environmental stresses [6], and it can grow in poor and acidic soils with low water availability [3,7] and high temperatures [8,9]. Nevertheless, production is always impaired to some extent by exposure to these stresses [7,10,11,12]. It is an excellent source of micro and macronutrients [13,14,15,16], supplying the need for essential amino acids when combined with cereals [17].

The world is going through climate changes that are critical for natural and agricultural ecosystems [7,18,19]. These changes reflect unrestrained human activity over decades [20] and are a capital threat to food security [21]. Hence, FAO aims to promote actions that contribute to ending hunger while not neglecting the protection of the environment, the planet, and its inhabitants, referring to legumes as crucial crops in this scenario [22,23,24]. The Mediterranean was one of the regions most prone to drought in the world between 1981 and 2010, and projections indicate that it will be equally or more severely affected in the coming decades [25,26]. In Europe, on average, 20% of the territory and 30% of the population is affected every year by water stress, with droughts causing considerable economic losses (2 to 9 billion Euros per year) and unquantifiable damage to ecosystems and their services [27]. Crop losses resulting from water deficit are much higher than those caused by other abiotic factors [28], and in this sense, the demand for drought-tolerant crops is increasing, becoming a priority in the current climatic setting. These pose new challenges in the management of existing genetic resources of crops, and in particular, pulses, for improved food and agriculture production.

Exposure to water deficit triggers many types of plant responses at the morphological, physiological, cellular, and molecular levels. These responses act in an integrated and complementary way [29,30], leading to escape, avoidance, and tolerance strategies that determine the ability of the plant to survive and produce under a water deficit. Yield is a complex outcome that results from the genotype background, environmental cues, and the interaction between both [31]. Several authors, including Iwuagwu et al. [32], reported that the effects of drought vary and depend on the intensity, duration of stress, development stage, strategy, and the plant’s adaptive capacity to cope with it. Nevertheless, a negative impact of water deficit on plant yield is inevitable, with water shortage also being responsible for possible changes in the quality and composition of the grain [33,34,35]. Some quality attributes, such as protein, fat, or carbohydrate content, may change [36,37]. Particularly in legumes, protein composition is very dependent on genetic background, but environmental factors, particularly drought, can also greatly influence protein levels in the grain. Studies report that depending on the legumes, protein content can decrease [36] or increase with the worsening of the water deficit [37,38].

Biodiversity is associated with greater resilience to environmental stresses [39], and the diversity of cultivated agricultural species is fundamental, both for achieving food security and for healthy and varied nutrition. Knowledge and preservation of varieties selected on-farm over many years may be the key to obtaining more resilient agri-food systems in the face of climate change. In this context, the present work aims to evaluate the impacts and responses of five Portuguese varieties of cowpea to terminal drought (imposed during the reproductive phase). We used four landraces of traditional significance from the central region of Portugal which are in danger of disappearing from the field, despite local efforts to keep them commercially available: L1 (BPGV13100) was kindly provided by the Banco Português de Germoplasma Vegetal (INIAV) and had been originally collected from Guarda; L2 was obtained directly from a farmer at Sátão; L3 was bought at a farmer’s market and is traditionally cultivated in the area of Lardosa; L4 was also obtained directly from farmers at Vila Maior. We also used a commercial variety, “Fradel”, developed at INIAV-Elvas under the Mediterranean climate to have improved characteristics for the farmers, such as an indehiscent pod and better productivity. The responses of plants to water deficit were evaluated in terms of development and morphological characteristics, as well as the impact of drought on the quality of the grain. Our aim is thus to characterize relevant cowpea Portuguese varieties, in terms of their resistance to water stress and its effect on nutritional characteristics, to preserve existing genetic resources and highlight their potential to develop improved varieties more resilient to climate change.

## 2. Materials and Methods

### 2.1. Plant Material and Growing Conditions

Seeds from a Portuguese commercial variety (CV) and four Portuguese Vigna unguiculata landraces (L1–L4) were sown in late May (Table 1) in numbered pots in a randomized distribution. Plants (1 plant per pot, 10 pots per landrace) were grown in a semi-controlled greenhouse and well irrigated to 80% of field capacity (FC) during the early vegetative growth. FC was evaluated by the gravimetric method. Pots were filled with ca. 3 L of peat moss soil (Arber Horticulture) and watered until saturation. After 24 h of runoff, saturation by capillarity was assured. Pots were weighed individually (approximately 1300 g), and the result is considered 100% FC [40]. Water deficit (WD) was induced in pre-flowering, 5 weeks after sowing, by withholding irrigation in half of the plants, maintained at 35% FC. Control plants (WW) were irrigated to maintain 80% FC during the entire phenological cycle of the plant. Once a week, water was replaced by a nutrient solution (Bayer Complesal NPK, 12-4-6) in both treatments.

**Figure 1:**
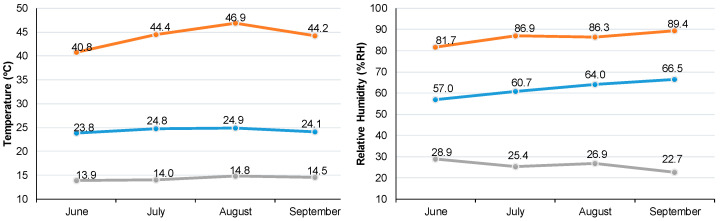
Monthly average values (blue), maximum (orange) and minimum (gray) temperature and relative humidity observed in the greenhouse during the test period.

Air temperature and relative humidity (%RH) were monitored with EasyLog USB Data Loggers (EL-SIE-2+, Lascar Electronics, Erie, PA, USA) during the whole plant growth cycle. The average, minimum, and maximum values of temperature and %RH are presented in Figure 1.

### 2.2. Phenological Development

Throughout the experiment, the phenological stage of each plant was evaluated according to the universal BBCH scale (Biologische Bundesantalt Bundessortenamt und CHemische Industrie) [41], with the main stages identified in this work described in Table 2.

### 2.3. Flowering and Pod Development

After the onset of the WD treatment at pre-flowering (T0), the number of open flowers on the main stem and secondary stems was registered weekly. The same was performed for the number of pods per plant (NPP) developed during the reproductive phase. The results are expressed as a cumulative average of flowers and pods per plant throughout the growth cycle for each genotype/treatment. In addition to T0, the results obtained two (T1), four (T2), five (T3), and six (T4) weeks after the onset of water deficit were evaluated.

### 2.4. Weight of 100 Grains

All grains from each plant were counted and weighed. The weight of 100 grains was calculated by extrapolation from Equation (1), where “W100G” corresponds to the mass of 100 grains, “*w_grains_*” to the mass of grains per plant (g), and “*n_grains_*” to the number of grains per plant.

(1) $$W100G=\frac{W_{grains}}{n_{grains}}\times 100$$

According to [42], this parameter allows classifying grains according to their size, as small (W100G < 25 g), medium (W100G between 25 and 40 g), and large grains (W100G > 40 g).

### 2.5. Colorimetric Analysis of the Grain

The grain color of each plant was determined by measuring the coordinates of the Commission Internationale de l’Éclairage—CIE system according to [43]. Briefly, color parameters, lightness (L), and chromaticity (coordinates a* and b*) were obtained with a Minolta CR 400 colorimeter (Minolta Co. Ltd., Osaka, Japan) coupled with a glass container for solid samples (CR-A504). Measurements were performed for illuminant D65 after calibration. L* measures the lightness of color and ranges from black (0) to white (100); a* indicates the contribution of red or green (when its value is positive or negative, respectively); and b* the contribution of blue or yellow (when its value is negative or positive, respectively). The elements of perceived color lightness (L*), Hue angle (h°), and chroma (C, saturation) were determined from the L* a* b* coordinates. Calculation of h° in degrees sets the color (starting at red, 0, yellow, up to 90, green, up to 180, and blue, up to 270). C* is a measure of color saturation or purity.

The results were expressed according to [43,44,45], where the total color difference ΔE is an index for total visible color differences and allows classification by categories: values between 0–0.5 (slightly discernible), 0.5 and 1.5 (difficult to detect by the human eye); 1.5 to 3.0 (visible difference, detectable by trained people) and 3.0 to 6.0 (appreciable differences detectable by ordinary people); large differences are already considered for values between 6.0 and 12.0 but within the same group of colors and values greater than 12 already represent extremes, with samples belonging to a group of different colors.

### 2.6. Soluble Sugars in Grain Determination

Soluble grain sugars were determined in approximately 400 mg of ground grain, based on the method of [46]. Briefly, the samples were homogenized in 10 mL of cold H_2_O, left to extract for 30 min on ice at 100 rpm, followed by 5 min in an ultrasonic bath and centrifuged (15,000× *g*, 20 min, 4 °C). The supernatant was collected, and the extraction procedure was repeated with the pellet. Both supernatants were combined and cleared with nylon syringe filters (0.45 µm) before injection. Sugars separation was performed with an HPLC system coupled to a refractive index detector (Model 2414, Waters, MA, USA), using a SugarPak 1 column (300 mm length × 6.5 mm diameter, Waters) at 90 °C, with H_2_O as eluent (containing 50 mg EDTA-Ca L^−1^ H_2_O) and a flow rate of 0.5 mL min^−1^. Standard curves were used for the quantification of each sugar.

### 2.7. Crude Protein in Grain

The protein content of ground grain samples was determined by the Kjeldhal method (NP1996:2000), through the quantification of the total nitrogen present in the samples, by adding 12.5 mL of sulfuric acid (95–97%), using potassium sulfate and selenium as catalyzers. Digestion took place at ca. 420 °C for at least 2 h. After cooling the sample at ambient temperature, distillation was performed to remove ammonia. Boric acid (4% *w*/*v*) and some drops of methyl red were added to the distillate prior to titration with hydrochloric acid 0.1 N. To determine the protein content in the samples, the results of the quantification of total nitrogen were multiplied by the conversion factor (6.25) based on the percentage of nitrogen in the protein [47].

### 2.8. Statistical Analysis

ANOVA (*p* < 0.05) was applied to these data using IBM SPSS Statistics 25 program (IBM SPSS, Inc., Chicago, IL, USA), followed by Tukey’s Test for mean comparison and regression analysis. Different letters express significant differences between landrace (a, b, c) or between WW and WD treatments for the same genotype (r, s). Bivariate analysis, using Pearson’s correlation, was also performed to assess the correlation between the analyzed parameters.

## 3. Results

### 3.1. Phenological Development

To access if the development of the five cowpea genotypes was differently affected by water stress, the main phases of the phenological cycle were annotated in the number of days after sowing (Table 3).

This analysis shows a similar duration of the vegetative phase in all genotypes (CV, L1 to L4), with the differences being verified only in the reproductive phase, both due to the influence of the genotypes and the imposed treatment (WW and WD). The commercial variety (CV) differed from the landraces right at the beginning of the reproductive phase (BBCH60), with anthesis (first flower) occurring about 10 days after the landraces (L1 to L4). At this stage, no differences were observed between treatments. As the reproductive phase advanced (BBCH80), a faster progression in the phenological cycle was observed for L1, L2, and L3 in response to drought, with the beginning of the maturation of pods happening 4 (L3), 7 (L1), and 15 (L2) days before the respective WW controls. The L2 variety maintained the strategy of anticipating the phenological state as a response to the drought, reaching the maturation of 80% of the pods (BBCH88) 13 days earlier than the control. This was not the case for L1 and L3, which progressively reduced the time difference between treatments. The imposed treatment did not affect the phenological development of L3 and L4 nor CV. It should be noted that CV, despite starting flowering later (BBCH60), reaches the remaining stages (BBCH80 and BBCH88) simultaneously with the controls of L1, L3, and L4.

### 3.2. Flowering and Pod Development

**Figure 2:**
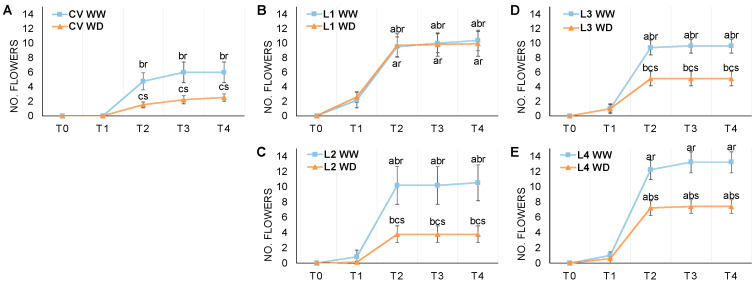
Flowering development (No. flowers) of Portuguese cowpea plants, a commercial variety (CV) (**A**) and four landraces, L1 (**B**), L2 (**C**), L3 (**D**), and L4 (**E**) under well-watered (WW) and water deficit (WD) conditions, at the beginning of treatment (T0) and 5, 7, 9, 10 and 11 weeks after, T1, T2, T3, and T4, respectively. Values represent mean ± SE (*n* = 5 to 10). Different letters mean significant differences between varieties (a, b, c) and between treatments for each variety (r, s). The letters (a) and (r) correspond to the highest values. (ANOVA, *p* < 0.05).

Under WW and WD conditions, CV showed a lower number of flowers than the landraces, 55%, and 38%, respectively. WD reduced the number of flowers in all genotypes, with the exception of L1, where the treatment had no impact on flowering. Drought did not affect the beginning of flowering in any of the genotypes (Figure 2), with greater precocity being observed in the landraces (in WW and WD) in relation to the CV, which blooms ca. 10 days later.

**Figure 3:**
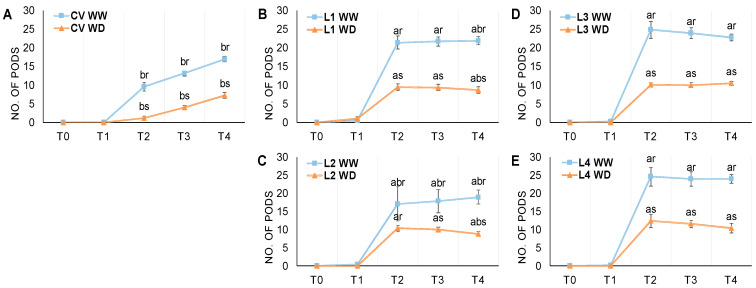
Pod development (No. of pods) of Portuguese cowpea plants, a commercial variety (CV) (**A**) and four landraces, L1 (**B**), L2 (**C**), L3 (**D**), and L4 (**E**) under well-watered (WW) and water deficit (WD) conditions, at the beginning of treatment (T0) and 5, 7, 9, 10 and 11 weeks after, T1, T2, T3, and T4, respectively. Values represent mean ± SE (*n* = 5 to 10). Different letters mean significant differences between varieties (a, b, c) and between treatments for each variety (r, s). The letters (a) and (r) correspond to the highest values. (ANOVA, *p* < 0.05).

In terms of pod development, pod onset occurred later for CV compared to landraces (Figure 3), in line with what was observed for the beginning of flowering. In CV, there was a gradual increase in the average of pods per plant between T2 and T4 (WW) and an average reduction of 72% of pods per plant in WD compared to the control.

On the contrary, for landraces, which showed a similar pattern among them, pod development occurred almost entirely between T1 and T2 (Figure 3).

### 3.3. Weight of 100 Grains

**Figure 4:**
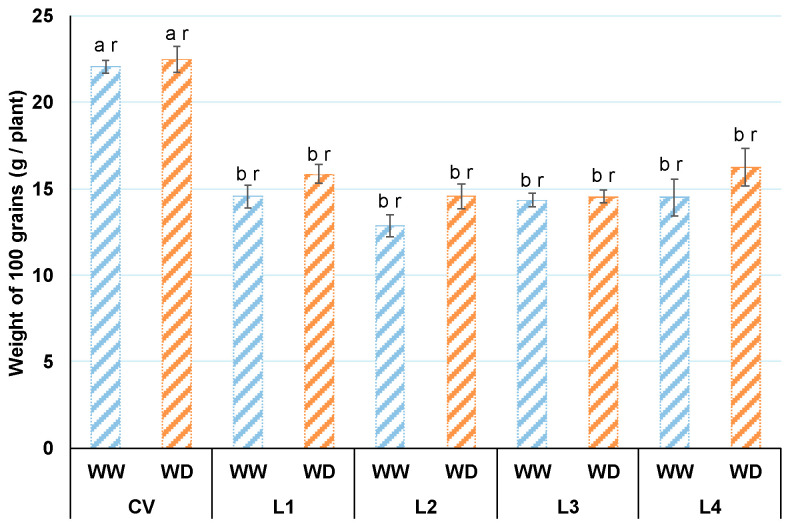
Weight of 100 grains (W100G) of Portuguese cowpea grains, a commercial variety (CV), and four landraces, L1, L2, L3, and L4, obtained under well-watered (WW) and water deficit (WD) conditions. Values represent mean ± SE (*n* = 5 to 10). Different letters mean significant differences between varieties (a, b, c) and between treatments for each variety (r, s). The letters (a) and (r) correspond to the highest values. (ANOVA, *p* < 0.05).

The imposed WD treatment did not result in differences in the weight of 100 grains (W100G) (*p* < 0.05) (Figure 4).

The CV produced the heaviest grains with W100G of around 22 g for both WW and WD. For the landraces, the W100G values ranged between 12.9 g and 16.2 g but with no significant differences among them.

### 3.4. Colorimetric Analysis of the Grain

The grain color of the four cowpea landraces and the CV were analyzed following CIE L* a* b* and CIE L* C* h° systems (Table 4) to access if the imposed treatment influenced the grain tegument pigmentation.

The values of L*, which range between black and white, show significant differences between all non-white genotypes, with the darkest colors presenting a value of 40.3 (WW) and 31.1 (WD) for the L4 variety and the opposite, corresponding to lighter colors values of 62.7 (WW) and 63.9 (WD) for the L3 and similar values for the white CV. The a* chromaticity scale which ranges between green and red, gave values close to zero for the white grains (CV and L3) and dark grains (L4). Varieties L1 and L2 present mean positive values indicating a slight movement towards more reddish colors. The b* chromaticity scale, which ranges between blue (negative) and yellow (positive), CV, L1, L2, and L3 presented close positive values corresponding to a slightly more yellow coloration than the dark L4 with lower values of b*. Analyzing the chromaticity value (C*), which represents the color saturation, describing more opaque or vivid colors, genotype L4 stands out as being the most opaque. By analyzing the hue angle (h°), genotype L4 also stands out as the only one expressively located between 90° (yellow hue) and 180° (gray-green hue), registering values of 103.9 (WW) and 129.3 (WD). In general, and taking into account the coordinates L* a* b*, we can observe that the variety most distinct from the rest in terms of color is L4, with significant differences (*p* < 0.05) for L* and b* compared to the other varieties, presenting a color closer to the black gamut. Considering the imposed treatment, no significant differences were verified for any of the above parameters, with the exception of a* in L2 (*p* < 0.05), for the CV, L1, L2, and L3, indicating that the water deficit did not have an impact on the color of these genotypes. The total color difference (ΔE), using WW grains as a control, corroborates these results. Varieties L1 and L3 had ΔE of 0.73 and 1.30, respectively, indicating slightly perceptible differences but difficult to distinguish by the human eye, and CV and L2 values suggest visible differences but only detected by trained people. The L4 variety showed a ΔE value of 10.84, revealing large differences in the same color group between treatments.

### 3.5. Soluble Sugars in Grain

The grain-soluble sugars were analyzed to access if different genotypes have different sugar content and also to determine whether the imposed WD had an impact on the accumulation of these compounds (Table 5). Under WW conditions, L2 presented the highest content of stachyose sugar (53.3 mg g^−1^) and L1 the lowest, 32.3 mg g^−1^. Under WD conditions, the lowest concentration was found in L3, 31.5 mg g^−1^, while CV had the highest concentration, 54.7 mg g^−1^.

WD caused different effects on the concentration of stachyose depending on the variety. L1 showed significant increases in the order of 62%, with the opposite occurring for L2 and L3, which showed significant reductions of 36% and 37%, respectively. On the other hand, WD did not cause a significant impact on the grain stachyose content in the CV and L4. In terms of raffinose, also a non-digestible sugar, the maximum values were observed for L4, both under WW and WD conditions, slightly above 10 mg g^−1^. The lowest raffinose content was recorded for L3 (4.0 mg g^−1^) under WW conditions and under WD conditions for L2 (4.2 mg g^−1^). In addition to L4, the imposed treatment also did not have an impact on the raffinose content of CV and L1. Quite differently, L2 had a reduction of 50% in the WD treatment, whereas L3 registered an increase of 218%. In the case of L3, the increase in raffinose was accompanied by a decrease in stachyose, while L2 had a reduction in both sugars. Glucose and fructose are the sugars in the lowest concentration, with percentages below 5%. Nevertheless, L1 e significantly accumulated these sugars under WD. Sucrose is the second most abundant sugar in cowpea, but with no differences between varieties, nor in response to the water treatments, with the exception of the L1 variety, which registered a slight (17%) but significant decrease under WD (Table 5).

The pattern verified for the most abundant sugar (stachyose) was also observed for the total quantified sugars. L1 showed an increase of 21%, and L2 a reduction of 30% in the WD treatment. The highest sugar content in the grain obtained from the control plants was registered for the landrace L2 (99.4 mg g^−1^), while under water deficit, CV registered the highest concentration of soluble sugars in the grain (104.6 mg g^−1^). The lowest concentrations of WW conditions were found for L1 (75.1 mg g^−1^) and under WD for L2 (69.3 mg g^−1^).

### 3.6. Crude Protein Content in the Grain

**Figure 5:**
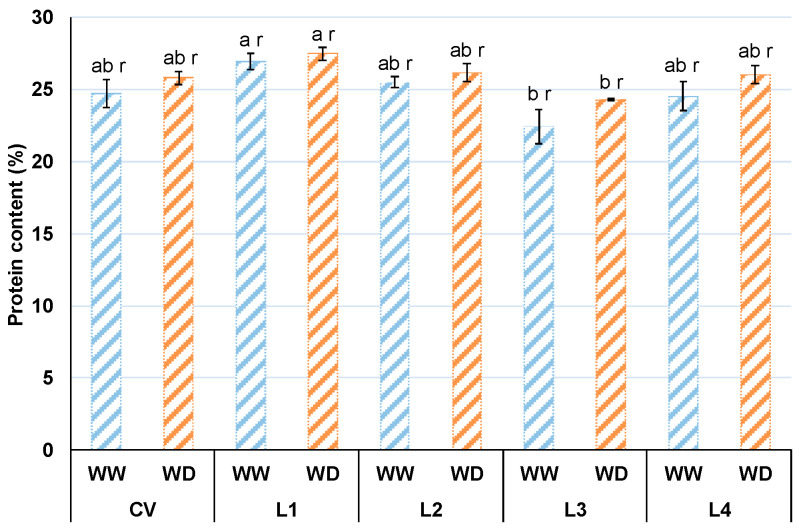
Protein content (%) in Portuguese cowpea grains of a commercial variety (CV) and four landraces, L1, L2, L3, and L4, obtained under well-watered (WW) and water deficit (WD) conditions. The values correspond to means (*n* = 3). Different letters mean significant differences between varieties (a, b, c) and between treatments for each variety (r, s). The letters (a) and (r) correspond to the highest values. (ANOVA, *p* < 0.05).

The obtained average crude protein content was quite stable for the genotypes under study ranging between 22 and 28% (Figure 5). The imposed water treatment did not impact crude protein content. Nevertheless, L1 presented the highest concentration, with average crude protein in the order of 27%, whereas L3 had the lowest values of about 24%.

### 3.7. Relationships between the Studied Agronomic, Physical, and Quality Parameters

The above-studied parameters were analyzed in terms of how they are affected by drought treatment and genotypes and the interaction of these two variables (Table 6). The parameters that are more variety-associated are the colorimetric parameters (L, a*, b*, C, h°) and the agronomic parameters W100G and NPP. On the other hand, the genotype did not significantly influence the contents of simple carbohydrates (sucrose, glucose, and fructose). The imposed water treatment had some effect on the colorimetric parameter b* (blue-yellow scale), chromaticity, oligosaccharides (stachyose and raffinose), crude protein, and W100G, particularly in NPP results. Considering the effect of treatment and variety together, sugars stachyose, raffinose, and the sum of the analyzed sugars, are the most significant. Crude protein, W100G and NPP did not show any significant effect of the treatment/genotype interaction.

Table 6 also shows the Pearson correlation values obtained for plants under WW and WD conditions.

Regarding the colorimetric parameters, the interpretation of the Pearson correlation values corroborates the analysis carried out for the ΔL and ΔC between the varieties, demonstrating a positive relationship between L and C. Therefore, lighter grain samples (L value higher) showed a more vivid color saturation (higher C value), or on the contrary, grain such as the L4 variety, darker (lower L value) appears more opaque (lower C value) (r = 0.762** WW; r = 0.845** WD). In turn, W100G showed a significant inverse relationship with NPP in both treatments (r = −0.335* WW; r = −0.568** WD), suggesting that plants producing a smaller number of pods have a higher W100G.

## 4. Discussion

Drought is a critical abiotic stress that strongly affects crop yields [36], including that of cowpea [48,49].

Plant growth and development are affected by drought, which can lead to reduced flower production and fewer grains, as well as constraints on grain filling and size [50,51,52]. Cowpea is reportedly more susceptible to water deficit during flowering and pod filling, resulting in flower abortion, pod drop, and reduced grain filling [53]. Morphologically, all genotypes suffered a rapid yellowing and fall of the adult leaves after the imposition of WD, leaving the available resources for the younger leaves, which remained green. This reduction in aerial biomass results in a decrease in the number of flowers and pods per plant obtained from each variety (Figure 2 and Figure 3). As suggested by Rollins et al. [54], the reduction in plant growth is a plant adaptation response to drought and not a secondary consequence of the decreased resources. It is the concerted relationship between phenological development and an efficient pattern of water use that allows a crop to adapt to stress, such as drought [55]. The flowering capacity and pod development of the studied varieties showed a similar outcome in response to stress, with a clear decrease in these two organs (Figure 2 and Figure 3), except for flowering in L1. Early flowering is reported as one of the main strategies to escape terminal drought in lentils and chickpeas [56,57], and a delay in flowering can cause great losses in crop yield [58]. In our study, the drought did not affect the beginning of flowering in any of the varieties (Figure 2). However, greater precocity was observed for the landraces (in WW and WD) relative to the CV, which blooms ca. 10 days later. This may reflect the greater hardiness and adaptation to hotter and drier climates of the four landraces. Unlike the other varieties, for L1, there was no difference in flowering between treatments (Figure 2, L1). However, these higher flowering values under drought did not translate into higher pod development between treatments (Figure 3, L1) due to the abscission of flowers during WD, preventing fruit set. Early maturation is referred to as an important strategy to avoid water deficit [59], having been observed in the L1 and L2 varieties, with L2 reaching 80% of pod maturation 13 days earlier compared to the WW control.

In addition to a crop’s ability to survive and withstand drought stress, the impact on yield potential and quality should also be considered [51]. One of the most important agronomic parameters for grain production in cowpea is the number of pods per plant, being associated with the yield potential [60,61,62], along with other characteristics such as the weight of 100 grains (W100G) [63].

Drought impact on the total number of pods per plant was evident (Figure 3) in all varieties, with an average decrease of 61%. The CV had the lowest number of pods for both treatments (20 pods per plant under WW conditions and six pods per plant under WD). However, these values were offset by the production of grains with higher mass (Figure 4). The decrease in production under WD for all the studied genotypes is clear, corroborating the concept that the stress occurring during the reproductive phase (from flowering to pod filling) has a great impact on cowpea productivity [64,65]. There were no significant differences in the W100G between the landraces nor between WW and WD conditions, showing that drought had no impact on this parameter in the studied Portuguese varieties. The decrease in grain filling may be associated with a reduction in the activity of starch synthesis enzymes and a resulting decrease in sucrose accumulation [50]. This does not seem to be the case in our cowpea landraces since they have maintained the W100G under drought. Deikman et al. [66] suggest that plant strategies/responses to stress, such as reduction in plant size and biomass, as well as low stomatal conductance, can largely explain the reduction in productivity.

Given their wide geographic distribution, cowpea is characterized by significant morphological variability and a great ability to adapt to different environments, which results in a wide range of landraces [67]. The nutritional composition of cowpea is the expression of genetic factors and the result of agroclimatic conditions, exposure to biotic and abiotic stresses, and harvest, post-harvest, and storage conditions [68,69,70,71]. The appearance of food is a key indicator of its quality and can determine its acceptance by the consumer [72]. Grain color is a key commercial characteristic. The diversity of grain colors is defined by the pigments and phenolic compounds present in the tegument. Factors such as variety, chemical composition, type, and duration of storage, among others, can influence grain color [42]. As expected from contrasting visual patterns, the analysis of variance confirmed the existence of significant differences (*p* < 0.05) between varieties for all colorimetric parameters. However, no significant differences were verified between WW and WD treatments for the CV, L1, L2, and L3 varieties, indicating that WD did not impact grain color. For L4, the analysis of variance showed significant differences between treatments for parameters L*, b*, C*, and h°, as well as a ΔE value of 10.84, revealing large differences in the same group of colors. However, this may be a consequence of the high standard error caused by the atypical phenotypic heterogeneity of the grains of this landrace and not an effect of WD.

The soluble sugar content of the grain is described as an important physiological/biochemical characteristic since it has an impact not only on production but also on the nutritional and cooking quality of the grain [13,73,74] and on their conservation and storage ability [75,76]. The increase in sugars in the grain has been related to desiccation tolerance during grain development [77], as it leads to osmotic adjustment, an important physiological response to drought in legumes [78]. Cowpea grain varieties have slightly different flavors based primarily on the type of present soluble sugars [76,79]. In general, cowpea grains are characterized by a high proportion of carbohydrates, which represent most of the dry weight of the grain, with the highest concentrations referring to starch and fiber. Additionally, the presence of eight soluble sugars (simple carbohydrates) is described, namely: sucrose (11–19 g/kg), glucose (4–5 g/kg), fructose (1–2 g/kg), galactose (≤15 g/kg), maltose (≤11 g/kg), and three sugars considered antinutrients, stachyose (17–60 g/kg), verbascose (6–13 g/kg), and raffinose (5–10 g/kg) [71]. In this study, the sugars quantification and profile (Table 5) presented values not far from those referred to in the literature, with stachyose oligosaccharide standing out, followed by sucrose, raffinose, glucose, and fructose. However, the sugar content variation under water stress is different among genotypes. CV and L4 showed no significant change, unlike their counterparts which suffered an impact of drought on the composition of the grain, mainly regarding the stachyose content, which increases in L1 under drought and decreases in L2 and L3. Cowpea has some constituents with antinutritional effects, including oligosaccharides (stachyose, raffinose, and verbascose) [80,81,82]. These antinutritional factors are chemical compounds synthesized by the plant in its defense against various stresses. In this context, the improvement of agronomic practices and stress control during production can contribute to the minimization of these disadvantageous factors [83]. Some studies have reported that drought tolerance is strongly correlated with the accumulation of non-reducing carbohydrates in the grain, mainly raffinose, stachyose, and verbascose [84]. The reduction in stachyose content in L2 and L3 under WD may be an advantage in terms of adverse effects on legume consumption. Sugars such as sucrose, glucose, and fructose can help improve the flavor of legume grains [85,86], with increased grain sweetness being reported to facilitate the acceptance and consumption of legumes such as cowpea [87]. The content of these readily available sugars showed little variability between varieties and treatments (Table 5).

The export rate of sucrose from the source (leaves) to sink regions (reproductive organs) depends on photosynthetic levels, with grain growth being supported by that sucrose export rate [88]. Previous work showed that leaf sucrose content did not differ between genotypes and did not change with WD [3]. The highest sugar content in the grain obtained under WW conditions was recorded for the landrace L2, which value decreased under WD. For L1, the inverse was observed, with sugars increasing under WD. Higher soluble sugar contents in the grain of cowpea varieties under drought conditions may be advantageous from a grain quality perspective [76].

Cowpea is an important alternative source of protein [89]. The quantification of grain protein and its variation with water deficit is a parameter that contributes to the assessment of the impact of drought on the nutritional quality of legumes [90]. The average crude protein content obtained in this work for landrace and commercial variety ranged between 22 and 28% (Figure 5), in line with that described by other authors, with results between 20 and 27% [17,71,91,92,93,94]. In Africa, taking advantage of its high protein content and the ability of the crop to withstand arid environments, cowpea consumption has been promoted in low-income families with the aim of reducing protein deficit and malnutrition [95]. Genetic diversity is responsible for variations in protein content [96], but for our genotypes, only L3 had lower protein content than its counterparts. WD had no impact on this parameter, thus showing maintenance of grain quality with stress contrary to what was reported by other authors, who refer to a decrease in protein content in legumes subjected to water stress [97], a consequence of decreased nitrogen uptake under limited water conditions [97,98].

Despite WD not having caused great differences in the studied variables (Table 6), assessing these results is still important to guarantee grain nutritional value and quality when obtained under stress.

## 5. Conclusions

The genotypes under study showed phenological adaptations to terminal drought. The evaluation of the physical and nutritional quality of the grain denoted little variation with the imposed water treatment, except for sugar levels from the raffinose family that is associated with the adaptive mechanisms of plants in response to drought. The preservation of the physical and nutritional quality of grains produced under drought indicates the advantage of using cowpea as a sustainable crop in drier environments.

The performance of the studied varieties confirms their adaptation to low water availability, a fundamental characteristic to face foreseen climate change without loss of grain quality. Landraces could contribute to more positive agricultural practices with lower environmental impact and higher food security outcomes or even increase the genetic variability for cowpea breeding programs.

## Figures and Tables

**Table 1:** Designation and origin of the cowpea genotypes under study.

Designation	Photo	Variety	Origin	Region	Main Features
CV	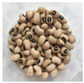	Commercial variety “Fradel”	INIAV—breeding program	Elvas— Alto Alentejo	Cream grain color, black eye around hilum, white flower
L1	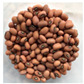	Landrace	BPGV * 13,100	Guarda— Beira Alta	Brown grain, small black eye around hilum, violet flower
L2	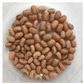	Landrace	Directly from the farmer	Satão— Beira Alta	Light brown grain color, small greenish-brown eye around hilum, violet flower
L3	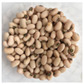	Landrace	Directly from the farmer	Lardosa— Castelo Branco, Beira Baixa	Grain is rounder than the other accessions, cream color and light green eye around hilum, white flower
L4	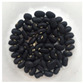	Landrace	Directly from the farmer	Vila Maior— Douro Litoral	Black grain, black eye around hilum, violet flower

* BPGV—“Banco Português de Germoplasma Vegetal”.

**Table 2:** Considered cowpea phenological stages according to the BBCH scale.

Growth Stage	BBCH Code	Description of the Growth Stage
Vegetative	05	Hypocotyl emergence
	20	Formation of secondary branches
	50	Inflorescence emergence
Reproductive	60	First flowers open
	80	Beginning of fruit and seed ripening
	88	80% of mature pods

**Table 3:** Analysis of the phenological development of Portuguese cowpea plants, represented by the number of days after sowing.

Variety	Treatment	DAS (28 May 2021)						
		Vegetative Phase				Reproductive Phase		
		BBCH 05	BBCH 20	BBCH 50		BBCH 60	BBCH 80	BBCH 88
CV	WW	4 ± 0 a	32 ± 0 a	39 ± 1 a	Water deficit imposition	60 ± 4 ar	77 ± 0 as	84 ± 0 ar
	WD					61 ± 2 ar	81 ± 1 ar	89 ± 3 ar
L1	WW	4 ± 0 a	32 ± 0 a	39 ± 0 a		50 ± 1 br	71 ± 4 ar	81 ± 1 ar
	WD					49 ± 1 br	64 ± 0 br	77 ± 1 bs
L2	WW	4 ± 0 a	32 ± 0 a	40 ± 1 a		52 ± 1 br	77 ± 0 ar	91 ± 6 ar
	WD					53 ± 1 br	62 ± 3 bs	78 ± 1 bs
L3	WW	4 ± 0 a	32 ± 0 a	40 ± 1 a		51 ± 1 br	73 ± 2 ar	81 ± 2 ar
	WD					51 ± 1 br	69 ± 2 abr	78 ± 1 br
L4	WW	4 ± 0 a	32 ± 0 a	40 ± 1 a		51 ± 1 br	77 ± 0 ar	81 ± 2 ar
	WD					51 ± 1 br	74 ± 3 abr	80 ± 2 br

CV—commercial variety; L1, L2, L3, L4—landraces of cowpea; WW—well-watered; WD—water deficit; DAS—days after sowing; Values represent mean ± SE (*n* = 5 to 10). Different letters mean significant differences between varieties (a, b, c) and between treatments for each variety (r, s). The letters (a) and (r) correspond to the highest values. (ANOVA, *p* < 0.05).

**Table 4:** Colorimetric analysis of Portuguese cowpea grains by the CIE L* C* h° and CIE L* a* b* system.

Variety	Treatment	Attribute				
		L*	a*	b*	C*	h°
CV	WW	60.1 ± 0.5 ar	−0.9 ± 0.1 cr	19.4 ± 0.5 abr	19.5 ± 0.5 ar	92.8 ± 0.4 abr
	WD	61.8 ± 0.7 ar	−0.4 ± 0.2 bcs	19.7 ± 0.3 ar	19.7 ± 0.3 abr	91.1 ± 0.5 bs
L1	WW	48.5 ± 0.7 cr	5.7 ± 0.5 ar	16.9 ± 0.6 br	17.9 ± 0.7 ar	71.6 ± 1.5 dr
	WD	48.7 ± 0.6 cr	6.3 ± 0.4 ar	17.3 ± 0.4 br	18.4 ± 0.5 br	70.2 ± 0.8 cr
L2	WW	53.7 ± 1.6 br	3.1 ± 0.4 bs	19.3 ± 1.4 abr	19.6 ± 1.4 ar	80.3 ± 1.8 cdr
	WD	53.1 ± 1.2 br	5.2 ± 0.5 ar	19.9 ± 0.8 ar	20.7 ± 0.7 ar	74.9 ± 1.9 cr
L3	WW	62.6 ± 0.3 ar	0.3 ± 0.2 cr	20.6 ± 0.2 ar	20.6 ± 0.2 ar	89.1 ± 0.5 bcr
	WD	63.9 ± 0.5 ar	0.6 ± 0.2 br	20.6 ± 0.2 ar	20.6 ± 0.2 ar	88.4 ± 0.5 br
L4	WW	40.3 ± 4.7 dr	−0.7 ± 0.7 cr	7.8 ± 2.7 cr	8.0 ± 2.5 br	103.9 ± 13.6 as
	WD	31.0 ± 0.1 ds	−1.9 ± 0.2 cr	2.3 ± 0.1 cs	3.0 ± 0.1 cs	129.3 ± 3.0 ar

CV—commercial variety; L1, L2, L3, L4—landraces of cowpea; WW—well-watered; WD—water deficit; L*—Lightness; a*—green (−) to red (+); b*—blue (−) to yellow (+); C*—Chromaticity; h°—hue angle. Values represent mean ± SE (*n* = 5 to 10). Different letters mean significant differences between varieties (a, b, c) and between treatments for each variety (r, s). The letters (a) and (r) correspond to the highest values. (ANOVA, *p* < 0.05).

**Table 5:** Soluble sugar contents (mg g^−1^) in cowpea grain in well-watered (WW) and water deficit (WD) conditions.

Variety	Treatment	Soluble Sugars in Grain (mg g^−1^)											
		Stachyose		Raffinose		Sucrose		Glucose		Fructose		Sum of Soluble Sugars	
CV	WW	48.6 ± 3.5	ar	9.0 ± 0.9	abr	27.9 ± 0.7	ar	2.9 ± 0.2	ar	2.8 ± 0.8	ar	91.3 ± 4.6	abr
	WD	54.7 ± 1.2	ar	10.5 ± 0.5	ar	32.5 ± 2.0	ar	3.9 ± 0.7	ar	3.0 ± 0.7	ar	104.6 ± 2.7	ar
L1	WW	32.3 ± 0.8	bs	7.1 ± 0.2	br	33.9 ± 1.8	ar	1.5 ± 0.2	as	0.4 ± 0.1	abs	75.1 ± 2.4	bs
	WD	52.3 ± 2.8	ar	6.2 ± 0.6	br	28.1 ± 1.2	as	2.7 ± 0.2	abr	1.9 ± 0.6	abr	91.1 ± 4.9	abr
L2	WW	53.3 ± 2.1	ar	8.4 ± 0.6	br	33.1 ± 2.1	ar	3.0 ± 1.1	ar	1.5 ± 0.9	abr	99.4 ± 5.4	ar
	WD	34.1 ± 1.6	bs	4.2 ± 0.4	bs	29.3 ± 1.6	ar	1.5 ± 0.2	br	0.2 ± 0.2	bcr	69.3 ± 2.4	cs
L3	WW	49.8 ± 2.6	ar	4.0 ± 0.4	cs	35.1 ± 4.5	ar	1.5 ± 0.5	ar	0.0 ± 0.0	br	90.4 ± 6.5	abr
	WD	31.5 ± 0.8	bs	12.7 ± 0.6	ar	31.0 ± 2.1	ar	2.5 ± 0.3	abr	0.0 ± 0.0	cr	77.7 ± 2.9	bcr
L4	WW	48.8 ± 1.5	ar	10.9 ± 0.4	ar	32.0 ± 2.8	ar	0.3 ± 0.1	as	0.0 ± 0.0	br	92.1 ± 4.3	abr
	WD	50.2 ± 0.7	ar	12.9 ± 1.1	ar	32.8 ± 1.8	ar	1.5 ± 0.1	br	0.0 ± 0.0	cr	97.4 ± 2.9	ar

CV—commercial variety; L1, L2, L3, L4—landraces of cowpea; WW—well-watered; WD—water deficit; Values represent mean ± SE (*n* = 3 to 5). Different letters mean significant differences between varieties (a, b, c) and between treatments for each variety (r, s). The letters (a) and (r) correspond to the highest values. (ANOVA, *p* < 0.05).

**Table 6:** Pearson correlation values and significance level of treatment, genotype, and their interaction on grain quality parameters.

Pearson Correlation	L*	a*	b*	C*	h°	Stachyose	Raffinose	Sucrose	Glucose	Fructose	Sum of Soluble Sugars	% Protein	W100G	NPP
L*	1	−0.099 ^ns^	0.885 **	0.845 **	−0.404 **	−0.264 ^ns^	−0.050 ^ns^	0.080 ^ns^	0.575 *	0.403 ^ns^	−0.129 ^ns^	−0.410 ^ns^	0.177 ^ns^	−0.253 ^ns^
a*	−0.496 **	1	0.329 *	0.411 **	−0.846 **	−0.209 ^ns^	−0.856 **	−0.518 *	−0.092 ^ns^	0.028 ^ns^	−0.492 *	0.395 ^ns^	−0.328 *	−0.305 *
b*	0.819 **	−0.034 ^ns^	1	0.996 **	−0.729 **	−0.303 ^ns^	−0.323 ^ns^	−0.217 ^ns^	0.506 *	0.409 ^ns^	−0.262 ^ns^	−0.179 ^ns^	0.010 ^ns^	−0.139 ^ns^
C*	0.762 **	0.071 ^ns^	0.993 **	1	−0.780 **	−0.302 ^ns^	−0.373 ^ns^	−0.245 ^ns^	0.490 *	0.405 ^ns^	−0.282 ^ns^	−0.137 ^ns^	−0.010 ^ns^	−0.112 ^ns^
**h°**	0.167 ^ns^	−0.880 **	−0.227 ^ns^	−0.308 ^ns^	1	0.290 ^ns^	0.728 **	0.456 ^ns^	−0.225 ^ns^	−0.249 ^ns^	0.452 ^ns^	−0.160 ^ns^	0.140 ^ns^	−0.120 ^ns^
**Stachyose**	0.441 ^ns^	−0.652 **	0.196 ^ns^	0.114 ^ns^	0.449 ^ns^	1	0.097 ^ns^	0.153 ^ns^	0.410 ^ns^	0.647 **	0.882 **	0.403 ^ns^	0.579 *	−0.490 *
**Raffinose**	−0.456 *	−0.194 ^ns^	−0.480 *	−0.496 *	0.289 ^ns^	0.208 ^ns^	1	0.504 *	0.160 ^ns^	−0.095 ^ns^	0.462 *	−0.408 ^ns^	0.283 ^ns^	−0.301 ^ns^
**Sucrose**	0.015 ^ns^	0.274 ^ns^	0.213 ^ns^	0.225 ^ns^	−0.258 ^ns^	0.065 ^ns^	−0.187 ^ns^	1	0.257 ^ns^	0.115 ^ns^	0.528 *	−0.195 ^ns^	0.354 ^ns^	−0.195 ^ns^
**Glucose**	0.473 *	−0.122 ^ns^	0.563 *	0.548 *	−0.027 ^ns^	0.286 ^ns^	0.102 ^ns^	0.046 ^ns^	1	0.830 **	0.571 **	−0.008 ^ns^	0.514 *	−0.485 *
**Fructose**	0.342 ^ns^	−0.208 ^ns^	0.332 ^ns^	0.313 ^ns^	0.070 ^ns^	0.164 ^ns^	0.372 ^ns^	−0.126 ^ns^	0.669 **	1	0.640 **	0.331 ^ns^	0.632 **	−0.561 *
**Sum of soluble sugars**	0.341 ^ns^	−0.441 ^ns^	0.252 ^ns^	0.190 ^ns^	0.282 ^ns^	0.862 **	0.332 ^ns^	0.445 ^ns^	0.459 *	0.348 ^ns^	1	0.157 ^ns^	0.633 **	−0.567 *
**% Protein**	−0.365 ^ns^	0.566 *	−0.077 ^ns^	−0.026 ^ns^	−0.441 ^ns^	−0.345 ^ns^	0.432 ^ns^	−0.305 ^ns^	0.044 ^ns^	0.034 ^ns^	−0.273 ^ns^	1	0.139 ^ns^	0.148 ^ns^
**W100G**	0.257 ^ns^	−0.357 *	0.078 ^ns^	0.059 ^ns^	0.209 ^ns^	−0.077 ^ns^	0.227 ^ns^	−0.337 ^ns^	0.216 ^ns^	0.536 *	−0.069 ^ns^	0.022 ^ns^	1	−0.568 **
**NPP**	−0.168 ^ns^	0.208 ^ns^	−0.164 ^ns^	−0.148 ^ns^	−0.088 ^ns^	−0.047 ^ns^	−0.220 ^ns^	0.274 ^ns^	−0.211 ^ns^	−0.382 ^ns^	−0.030 ^ns^	0.022 ^ns^	−0.335 *	1
**Significance level**														
**Treatment**	ns	ns	*	*	ns	*	*	ns	ns	ns	ns	*	*	***
**Genotype**	***	***	***	***	***	***	***	ns	*	**	**	**	***	***
**Genotype × Treatment**	*	*	*	*	*	***	***	ns	*	ns	***	ns	ns	ns

L*—Lightness; a*—green (−) to red (+); b*—blue (−) to yellow (+); C*—Chromaticity; h°—hue angle; W100G—Weight of 100 grains; NPP—Number of pods per plant. The upper diagonal in salmon represents plants grown under water-stressed conditions, and the lower diagonal in blue represents plants grown under well-watered conditions. Values close to zero indicate the absence of correlation, and values close to one indicate a strong correlation between two parameters. Orange represents the effects of treatment, genotype, and their interaction in the analyzed parameters, being: ns—not significant, *—significant (*p* < 0.05), **—very significant (*p* < 0.01), ***—highly significant (*p* < 0.001).

## Data Availability

Not applicable.
